# Exploring the effect of biological delays in kinetic models of influenza within a host or cell culture

**DOI:** 10.1186/1471-2458-11-S1-S10

**Published:** 2011-02-25

**Authors:** Benjamin P Holder, Catherine AA Beauchemin

**Affiliations:** 1 Department of Physics, Ryerson University, Toronto, ON, M5B 2K3, Canada

## Abstract

**Background:**

For a typical influenza infection in vivo, viral titers over time are characterized by 1–2 days of exponential growth followed by an exponential decay. This simple dynamic can be reproduced by a broad range of mathematical models which makes model selection and the extraction of biologically-relevant infection parameters from experimental data difficult.

**Results:**

We analyze in vitro experimental data from the literature, specifically that of single-cycle viral yield experiments, to narrow the range of realistic models of infection. In particular, we demonstrate the viability of using a normal or lognormal distribution for the time a cell spends in a given infection state (e.g., the time spent by a newly infected cell in the latent state before it begins to produce virus), while exposing the shortcomings of ordinary differential equation models which implicitly utilize exponential distributions and delay-differential equation models with fixed-length delays.

**Conclusions:**

By fitting published viral titer data from challenge experiments in human volunteers, we show that alternative models can lead to different estimates of the key infection parameters.

## Background

In the past decade, mathematical models of viral infection have been successfully applied to a number of problems on the periphery of the annual public health problem that is influenza [1]. In the laboratory, mathematical models have aided the development of efficient vaccine production techniques [2] and improved the quantitative characterization of antiviral drug action [3]. Mathematical models have also improved our understanding of the course of the disease within human [4] and animal hosts [5]. Because these models serve as a bridge between the microscopic scale (where virus interacts with cell) and the macroscopic scale (where the infection is manifested as a disease) they will inevitably be applied in the future to pressing public health questions such as the estimation of virulence and fitness for emerging strains, the spread of drug resistance and, more generally, the connections between viral genotypic information and clinical data.

The success of a within-host virus infection model depends on an accurate representation of biological reality. This allows a model not only to describe the phenomenon under consideration, but also to make reliable predictions about unobserved consequences. For example, in 1995 a simple model of HIV dynamics was applied to describe the observed exponential clearance of virus under the administration of a drug suppressing viral production [6]. The primary result of this work, however, was not the description of viral clearance itself, but the prediction of dynamics in the absence of drug, i.e., that high viral clearance must be balanced by high viral production, which in turn allows for extremely rapid mutation of the virus strain. This conclusion had important implications for the development of therapy, specifically the necessity of a “drug cocktail”. For influenza infections, the primary clinical data available to a mathematical modeler is the viral titer over the course of an infection, usually obtained by a daily nasal wash collected from an infected patient. This data generally follows a simple functional form in time which can be reproduced by a variety of dynamical models. Thus, if meaningful information is to be extracted from such data, the model applied must already be a trusted simulator of the underlying infection kinetics. In this paper, we consider evidence from laboratory infection experiments which must inform the construction of a mathematical model, focusing specifically on the implementation of the time spent by a cell in each of the various stages of infection.

**Figure 1:**
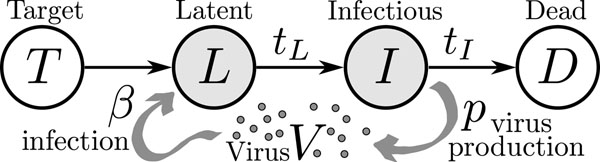
**Basic viral infection model**. The cell-virus interactions of infection (*β*) and viral production (*p*) are indicated along with the four possible states in which cells can be found. The lifetimes of the latent and infectious states for a particular cell, *t_L_* and *t_I_*, are considered random variables.

The basic viral infection model [4,7] assumes interaction of virus with cells in four different states (Figure 1), and is based on a coarse-grained view of the virus replication cycle. Cells that have not yet been infected by the virus, but are susceptible to infection, are considered target cells (*T*). The interaction of virus with target cells leads to these cells becoming latently (*L*) infected (i.e., infected but not producing virus). After infection, a time, *t_L_*, passes — as the virus particle is unpacked, its genome is delivered to the cell nucleus, replication begins, and new particles assemble at the plasma membrane — before new virus particles are released and the cell enters the infectious (*I*) state. After a subsequent time, *t_I_*, the infectious cell halts virus production and transitions into a state we will refer to as dead (*D*).

The implementation of a particular dynamical structure on this basic model requires a more detailed specification of the biological processes. The infection of cells (the transition of target cells to latently-infected cells) has been observed to be a Poisson process where the rate of infection is proportional to the local virus concentration [8] and it is implemented in the model as a continuous representation of that stochastic process. Virus production by infectious cells can be assumed to proceed at a constant rate and the infectivity of free virus is known to decrease exponentially in time [3,9], leading to a simple equation for virus dynamics. To complete the dynamical description, one must specify how a latently infected cell becomes infectious and for how long infectious cells produce virus. In other words, one must specify the distribution of the delays, *t_L_* and *t_I_*, between the states of infection.

In an epidemiological context, the problem of implementing generic delays between infected classes was first considered by Kermack and McKendrick in their seminal 1927 work on infectious disease dynamics [10]. Hethcote and Tudor [11] introduced a general approach to the problem, using a probability density function for the time spent in a given state, which has been applied frequently in the field of mathematical epidemiology (see, e.g., [12-14] and references therein). Here, we will apply the same approach to within-host influenza viral infections, resulting in a model with differential equations to describe target cell and virus dynamics, and integral equations to describe the latent and infectious cell populations (a similar approach was considered for HIV in [15]).

**Figure 2:**
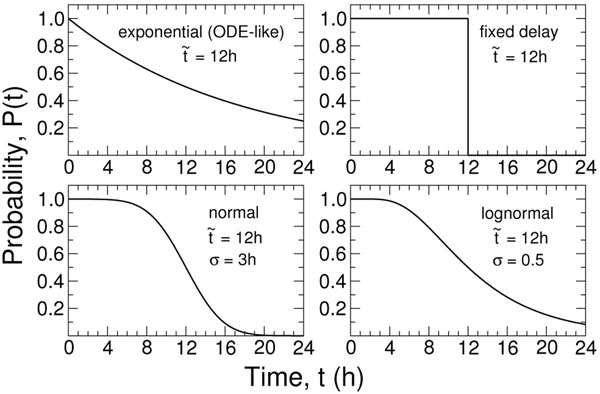
**Example delay distributions**. Four example delay types defining the time spent by a cell in a given state. The plotted function, *P*(*t*) corresponds to the probability that after some time *t*, the cell remains in its current state, i.e., has not yet transitioned to the next state. In each example,  is the median time of transition to the next state. While exponential-delays allow for instantaneous transitions (e.g., 10% of cells will have transitioned after 1.5 h has elapsed), the other three types enforce a minimum waiting period for almost all cells before transitioning from one state to the other (i.e., *P*(*t*) *≈* 1 for 0 *< t < t*_min_). Enforcing this delay is more biologically realistic. The function *P*(*t*) appears explicitly in the viral infection model, Equation (1), and allows for generic delays between states of infection.

Mathematically, the simplest choice of delay distribution is exponential (shown in Figure 2 with a few other choices), because it reduces the model to a system of ordinary differential equations (ODEs). For that reason, it is the most commonly-used model type for both epidemiological and within-host problems. In viral infections, however, the assumption of an exponential distribution seemingly conflicts with the biological evidence. For example, if the time of latent infection is chosen from such a distribution, the model would predict that a significant fraction of cells begin producing virus almost immediately after infection. In reality, however, there is always a minimum delay prior to viral release: endocytosis and the fusion of the viral envelope with the endosome takes, on average, half an hour [16]; the viral RNA enters the nucleus in most cells within the next one hour [17]; mRNA is transcribed in the nucleus, then transported back to the cytoplasm for translation and newly formed M1 matrix proteins are observed only three hours after infection, on average, and hemagglutinin four hours post-infection [17]; newly formed glycoproteins, matrix proteins and nucleocapsids then must assemble at the cell membrane, bud off and be cleaved from the sialic acid receptors [18]. Each of these steps and their timings depend on virus strain and cell type, and one can expect significant variation between cells, but a long delay without viral production is an essential characteristic of the infection cycle. Influenza virus-induced cell death is less well characterized: the mechanism of cell killing (apoptosis or necrosis) depends on cell type [19,20], and the timing of apoptosis in particular is strongly strain dependent [21]. In this situation, a broad freedom in selecting the distribution for infectious cell lifespans is warranted.

Despite questions about their biological appropriateness, ODE models have had success in describing in vivo infection data (for influenza see, for example, [4,5]). Models with non-exponential delays have been similarly successful, including those with Dirac delta function transition distributions, leading to a delay-differential model [3,4,22]; and multi-compartmental ODE models (with *n* sequential phases of infection) yielding delays with a gamma-function distribution [23-25] Here, we consider a set of in vitro experiments which allows for some discrimination between models, namely the single-cycle viral yield assay. By fitting models with different transition distributions (Figure 2) to single-cycle assay data, we show that the correct implementation of delays is crucial to the success of a model in describing these assays. Using these results, we consider in vivo data from challenge experiments in humans to explore how the choice of delays affects the parameter values extracted when fitting the model to experimental data.

## Methods

### Model

The general viral infection model used in this paper is written, following [11], as(1)

where *T*, *L*, *I* are the populations of cells in the target, latently-infected and infectious (virus-producing) states, respectively, and *N* the total number of cells in the system. *V* is the virus concentration, *β* and *p* are the rates of infection and virus production, respectively, and *c* is the viral clearance rate. The function *P_L_*(*t*) is the probability that a cell remains in the latent state for at least a time *t* before transitioning to the infectious state, and *P_I_*(*t*) is the probability that a cell remains in the infectious state for at least a time *t* before transitioning to the dead state (i.e., before it ceases to release virus). The transition profiles for different choices for the expression of *P*(*t*) are illustrated in Figure 2. *f_L_* is the probability density function for the time a cell will spend in the latent state before transitioning to the infectious state (*f_L_* = *–*d*P_L_/*d*t*). Note that *f_I_*(*t*) does not explicitly appear in the model.

### Delay distributions

If an exponential distribution is chosen for both transitions (*exponential-delay*), we have(2)

where *τ_L_* is the average time spent by a cell in the latent infection state, and *τ_I_* is the average lifespan of an infectious cell. Equations (1a–1d) can then be written as a system of ordinary differential equations(3)

When a Dirac delta function is used for both *f_L_* and *f_I_* (*fixed-delay*), such that the times spent by cells in the latent state and the infectious state are exactly *τ_L_* and *τ_I_*, respectively, Equations (1a–1d) reduce to a set of delay differential equations (DDE) [3].

Biologically, cells transition from one state to another only after some average time has elapsed. One also expects to see some variation in these transition times among cells as many of the processes rely on chains of stochastic intracellular events. Thus, normal distributions,

where *σ_L_* and *σ_I_* are the standard deviations, are a natural choice (*normal-delay*). The normalization constants, *A_i_*, are necessary since the distributions must be truncated at zero. If one wishes to avoid renormalization, a good alternative is a lognormal distribution (*lognormal-delay*)

where erfc(*x*) is the complementary error function (i.e., erfc(*x*) = 1 – erf(*x*)) and the standard deviations *σ_L_* and *σ_I_* are dimensionless quantities.

To facilitate comparison between the various distributions, we utilize their median values. For the Dirac delta and lognormal distributions, the median is simply the value of the parameter itself, i.e.,(4)

For exponential distributions, the median is(5)

and for the truncated normal, the median is found by setting its cumulative distribution function to one-half (there is no simple analytical expression).

### Numerical simulation

Numerical evaluation of the model in Equation (1) was performed using a modified Euler technique. At every time step of length ∆*t*, newly infected cells, *L*_new_ = *βTV* ∆*t*, were removed from the target population. The passage of these *L*_new_ cells through the latent and infectious states was then calculated for all future times using *f_L_*, *P_L_* and *P_I_* and added to that of previously transitioned cells. Virus dynamics at each time step were calculated according to the Euler approximation of Equation (1d). Simulations of single-cycle in vitro experiments were initialized with *L*(0) = *N*; simulations of in vivo infections were initialized with *T*(0) = *N* and *V* (0) = *V*_0_.

### Model fitting and parameter extraction

In any model-fitting exercise, a number of considerations must be made to ensure the reliable extraction of parameter values from experimental data. First, one must consider the question of parameter identifiability [36,57,58]: If the experimental system were to exactly reproduce the dynamics of the model equations, could the parameters be uniquely identified from the available observations? A number of techniques have been introduced to address identifiability for ODE models [37,59], but these are not directly applicable to the more general system considered here (Equation (1)). Nevertheless, for each experiment considered here we attempted to reduce consideration to an identifiable set of parameters. For the early phase single-cycle viral yield experiments, we fixed parameter values involving viral clearance and infectious cell death, and fitted only those parameters related to viral production and the transition of cells from the latent to infectious state. In the single-cycle, single-history experiment, independent information on viral clearance allowed for that parameter to be fixed in fitting. For the in vivo volunteer patient infection data, we have arbitrarily fixed the product of the viral production and infection rates in order to obtain a unique solution in the fitting procedure.

A second consideration when fitting experimental data is the question of model error: Is the mathematical model an appropriate representation of experimental data? To address this question, we performed least squares fitting of the model equations to the data sets using the Octave 3.2.4 [60] implementation of the Levenberg-Marquardt algorithm, leasqr. For all data presented here, fits were performed to the viral titer data in log-space and the sum of squared residuals (SSR) was calculated as 

In order to compare model systems with different numbers of parameters, we evaluated the Akaike information criterion (AIC) for each fit,(6)

where *k* is the number of model parameters, *n* is the number of data points and AIC_c_ is the “corrected” form of the AIC for small sample sizes [61].

Finally, to account for measurement error, we calculated 95% confidence intervals for each reliably extracted parameter value by fitting 1000 bootstrap replicates [62]. Confidence intervals were not calculated for models fits with large error (high SSR and AIC_c_) or for fits to volunteer patient infection data, where arbitrary assumptions were made to constrain the fitted parameter values.

### Calculation of the basic reproductive number

For each fit to the volunteer patient infection data we numerically calculated an approximation of the basic reproductive number, *R*_0_. In the ODE model, Equation (3), linear stability of the disease-free equilibrium is guaranteed by *R*_0_*<* 1, where(7)

It can also be shown using the ODE that this quantity is equal to the commonly-quoted definition of the reproductive number: *the number of secondary infections caused by one infectious cell, in a completely susceptible cell population*. For other delay models, where an analytical form is not readily available, we calculated *R*_0_ numerically according to that statement, i.e., for a given set of parameters, we disallowed latent to infectious transitions, initialized the simulation with one infectious cell, and determined the number of cells in the latent state as *t → ∞*.

## Results and discussion

### General features of single-cycle viral growth

Single-cycle growth (SCG) viral yield experiments provide a unique view of viral replication. By initiating infection with a viral inoculum of high concentration (a multiplicity of infection (MOI) much larger than one), all cells are infected simultaneously, and the experimentalist effectively synchronizes the cells’ passage through the phases of latency, viral production and death. The resulting viral production curve can then be viewed as that of the average cell. This is in sharp contrast to “multiple-cycle” yield experiments (MOI ≪ 1), where only a few cells are initially infected, leading to successive cycles of infection; the resulting exponential growth of both infected cells and virus over time effectively masks the dynamics of a single cell. Virus infections of humans and animals are similar to this latter experiment in that they are likely initiated by the infection of a only a few cells [26,27], leading to the exponential consumption of a large target cell population. Thus, SCG experiments demonstrate an artificial infection dynamic which would never occur in nature. However, their depiction of the average virus production of a cell makes them an invaluable tool for model building and for isolating specific components or parameters of the viral replication cycle.

**Figure 3:**
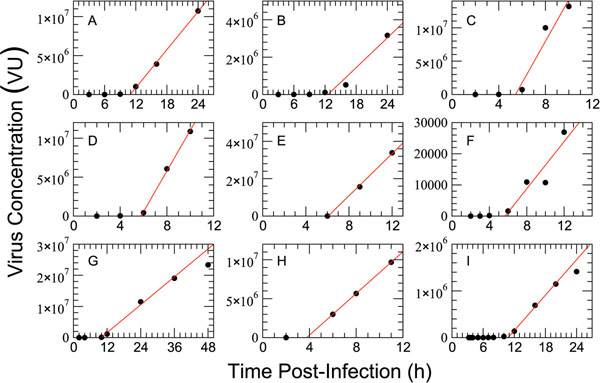
**Single-cycle viral yield examples.** Examples of single-cycle growth experiments. Plotted lines are fits to the non-zero data points using Equation 8 (the last data point in **G** and **I** have been excluded from the fits) Virus units (VU), the parameters of the fit, and the details of each experiment are given in Table 1.

In the original publications of the nine example SCG data sets plotted in Figure 3[9,28-32] virus was plotted on a logarithmic scale, due to the extraordinary sensitivity of measurement techniques which can detect infectious virus over nearly 8 orders of magnitude. It is clear from Figure 3, however, that this perspective masks the most important features of SCG: a long delay without significant viral release followed by linear growth of the virus concentration. This simple dynamic can be summarized in the empirical expression,(8)

where  and p* can be considered approximations of the average latent infection period, *τ_L_,* and the viral production rate, *p,* in the case where viral clearance is low (i.e., Equation (8) is the solution of Equation (1d) when *I/N* = 1 and *c* = 0). The fitted values of  and p* for all nine experiments are given in Table 1.

**Table 1:** Information on the individual single-cycle viral yield experiments plotted in Figure 3

(Label) Strain	Cell Type	MOI^a^	VU^b^	p* (VU/h)	(h)	ref.
(A) A/Udorn/307/72 (H3N2)	A549	3	PFU/mL	(8.2 *±* 0.3) *×* 10^5^	10.9 *±* 0.8	[28]
(B) A/Udorn/307/72 (H3N2)^c^	A549	3	PFU/mL	(2.7 *±* 0.6) *×* 10^5^	12 *±* 5	[28]
(C) A/Udorn/307/72 (H3N2)	A549	5	PFU/mL	(3.1 *±* 0.9) *×* 10^6^	(6 *±* 3)	[29]
(D) A/Udorn/307/72 (H3N2)^d^	A549	5	PFU/mL	(2.6 *±* 0.1) *×* 10^6^	5.8 *±* 0.4	[29]
(E) A/Udorn/307/72 (H3N2)	MDCK	5	PFU/mL	(5.6 *±* 0.3) *×* 10^6^	(6.1 *±* 0.4)	[29]
(F) A/PR/8/34 (H1N1)	MDCK	10	TCID_50_/mL	(4 *±* 1) *×* 10^3^	6 *±* 3	[30]
(G) A/PR/8/34 (H1N1)	MDCK	32	PFU	(7.5 *±* 0.4) *×* 10^5^	(9.8 *±* 1)	[9]
(H) A/X-31 (H3N2)	Vera	50	TCID_50_/mL	(1.34 *±* 0.01) *×* 10^6^	(3.75 *±* 0.01)	[31]
(I) A/NWS/33 (H1N1)	1-5C-4	50	PFU/mL	(1.26 *±* 0.07) *×* 10^5^	10.8 *±* 1	[32]

a — The number of cells used in each experiment was ~ 2 *×* 10^6^ except for (F) and (G) which had 9 *×* 10^6^ and 3 *×* 10^5^ cells, respectively.

b — Virus Unit. It should be noted that this unit is inherently experiment-specific, depending on virus strain, cell type and experimental details. Quantities which depend on VU (e.g., p*) cannot be directly compared between experimental groups.

c — NS1-T215A recombinant mutant.

d — NS1-R38A recombinant mutant.

We will consider below the characterization of the SCG experiment using various dynamical models, but some simple analysis can be done using only the empirical relation above. For example, two experiments (Figure 3A-B and Figure 3C-D) considered the growth of influenza A/Udorn/307/72 (H3N2) and a counterpart strain possessing a single mutation in the NS1 gene (T215A and R83A, respectively) [28,29]. In each case, the experiment reveals a significant reduction of the approximate viral production rate p* for the mutant, without a significant change in the approximate latency period. This shows that the single-cycle experiment can highlight important biological characteristics of a virus strain, with very little mathematical analysis.

### Characterizing the latent infection period from single-cycle growth assays

**Figure 4:**
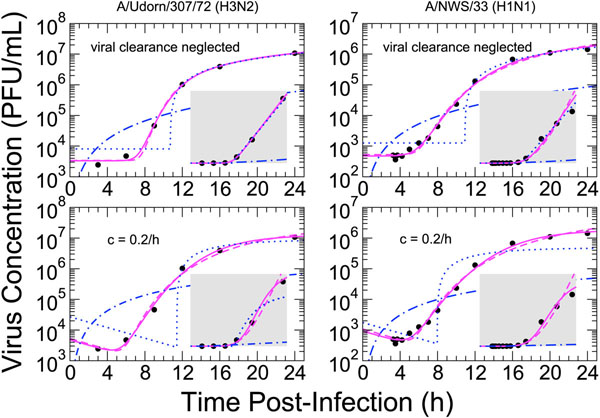
**Model fits to viral titers from single-cycle experiments**. Model fits to viral titers from single-cycle experiments by (Left) Hale et. al. [28] (Figure 3A) and (Right) Sugiura and Kilbourne [32] (Figure 3I). Fitting was performed using an exponential (dot-dashed), delta (dotted), normal (solid) or lognormal (dashed) distribution for the time spent by newly infected cells in the latent state before transitioning to the infectious state. Insets show the same fits and data on a linear virus scale (axis ranges are identical to those in Figure 3).

To determine the type of distribution which should be used to model the time spent by a newly infected cell in the latent phase, we performed model fits to two single-cycle data sets [28,32] which offered frequent sampling in time and a large range of virus measurements (Figure 4). Recently we have shown [33] that the ODE assumption of exponential delays yields a very poor fit to single-cycle viral yield data. Here, we considered three additional distributions for the waiting times: fixed-delay, normal-delay and lognormal-delay. We neglected the influence of infectious cell death by setting the infectious cell lifespan to be much longer than the duration of the experiment. This is an oversimplification of reality, particularly for later times. However, the lack of data spanning the period of virus decline, which occurs at later times than those measured, prohibits any real measure of infectious cell death. Similarly, this lack of viral decay information prohibits an effective characterization of the loss of virus infectivity. Therefore, we fitted the model using two values of viral clearance, zero and 0.2 h^–1^. The latter value represents an approximate upper-bound of the viral clearance value based on literature reports for in vitro experiments performed at 37°C [2,3].

The results of model fits to the two data sets are collected in Table 2. The exponential- and fixed-delay models clearly provide a poorer fit to the data than those with normal- and lognormal-delays (for the A/Udorn/307/72 [28] data set, however, the small number of data points and fewer parameters, with respect to normal and lognormal models, do not allow for their formal exclusion based on AIC_c_). While the exponential-delay model (ODE) is unable to duplicate any feature of the dynamics, the fixed-delay model fits the data well at late times in the infection when the dynamics are dominated by linear growth, at least when viral clearance is neglected. It does a poor job, however, of describing the early stages of virus release, and is unable to provide the correct dynamics in the presence of viral clearance.

**Table 2:** Fits to two single-cycle viral yield experiments (Figure 4)

A/Udorn/307/72 (H3N2) [28]								
Dist^a^	*c*(h^–1^)			*τ_L_* (h)	*σ_L_*	(h)	SSR	AIC_c_

exp	(0)^b^	0	2.4 *×* 10^6^	1200	—	830	4.86	16.7
*δ*	(0)	8.1 *×* 10^3^	7.7 *×* 10^5^	10.7	—	10.7	0.90	6.6
*N*	(0)	3.3 *×* 10^3^	8.3 *×* 10^5^	11.1	1.5 h	11.1	0.039	17.8
		[2.5:4.4]^c^	[5.9:12.7]	[9.8:12.5]	[0.8:2.2]			
ln*N*	(0)	3.4 *×* 10^3^	8.2 *×* 10^5^	10.9	0.15	10.9	0.041	18.1
		[2.4:4.1]	[5.6:12.8]	[9.7:12.6]	[0.08:0.22]			
exp	(0.2)	0	10^36^	10^32^	—	10^32^	5.79	17.8
*δ*	(0.2)	27000	1.8 *×* 10^6^	11.4	—	11.4	1.73	10.5
*N*	(0.2)	4.8 *×* 10^3^	2.6 *×* 10^6^	14.0	2.7 h	14.0	0.068	21.1
		[3.1:7.7]	[1.8:4.4]	[13.0:15.9]	[2.3:3.3]			
ln*N*	(0.2)	5.3 *×* 10^3^	5.2 *×* 10^6^	19.3	0.37	19.3	0.144	25.6
		[2.8:10]	[1.7:11.0]	[12.5:57.9]	[0.22:0.58]			

A/NWS/33 (H1N1) [32]								

Dist	*c*(h^–1^)			*τ_L_* (h)	*σ_L_*	(h)	SSR	AIC_c_

exp	(0)	0	1.4 *×* 10^26^	4.8 *×* 10^23^	—	3.3 *×* 10^23^	8.30	2.8
*δ*	(0)	1300	1.2 *×* 10^5^	10.9	—	10.9	2.77	-11.4
*N*	(0)	4.7 *×* 10^2^	1.5 *×* 10^5^	12.7	2.5 h	12.7	0.090	-51.7
		[3.8:5.7]	[1.1:2.2]	[11.5:14.1]	[2.1:3.0]			
ln*N*	(0)	5.3 *×* 10^2^	2.3 *×* 10^5^	15.3	0.33	15.3	0.167	-43.6
		[4.1:6.6]	[1.1:9.2]	[11.6:26.1]	[0.24:0.47]			
exp	(0)	0	4.5 *×* 10^31^	9.1 *×* 10^28^	—	6.3 *×* 10^28^	11.0	6.5
*δ*	(0.2)	1.8 *×* 10^3^	9.6 *×* 10^4^	7.9	—	7.9	2.11	-15.0
*N*	(0.2)	9.3 *×* 10^2^	3.8 *×* 10^5^	14.4	3.0 h	14.4	0.099	-50.4
		[3.9:11]	[1.1:5.9]	[11.6: 21.9]	[0.3:3.3]			
ln*N*	(0.2)	1.1 *×* 10^3^	1.2 *×* 10^6^	23.8	0.44	23.8	0.22	-40.0
		[0.79:1.4]	[0.37:6.0]	[15.3:109]	[0.32:0.71]			

a — Distribution used: exponential (exp), Dirac delta (*δ*), normal (*N*), and lognormal (ln*N*).

b — Values in parantheses were held fixed

c — 95% confidence intervals are given in brackets for the normal and lognormal fits.

Both the normal- and lognormal-delay models provide an adequate description of the data over the entire range of values and for both values of viral clearance, although the SSR and AIC_c_ values are smaller for fits using a normal distribution. When the fits to the log-valued virus are viewed in linear-space (inset graphs), the normal fits appear to be a more reasonable approximation of the data in that the linear SSRs of these fits, which depend most sensitively on the larger virus values, are also smaller. When viral clearance is neglected, the fitted values of the viral production rate, *p,* are close to the approximate values of p* (Table 1). Non-zero viral clearance leads to a larger fitted production rate, as expected. The fitted values for the median latent infection period, , vary depending on the distribution type, but are always as long as the approximate values of  for both experiments (10.9 h for [28] and 10.8 h for [32]). The introduction of a non-zero viral clearance leads to even longer latent infection periods, ranging from 8 h to 24 h. The fitted standard deviations, *σ_L_*, are between 1.5 and 3.0 h for the normal distribution and between 0.15 and 0.44 for the lognormal distribution.

### Characterizing the infection cycle from a single-cycle, single-history yield assay

In 1968, an in vitro experiment was performed which, to our knowledge, is unique in the literature [9]. Like the experiments presented in the previous sections, a SCG experiment was prepared: ~ 10^7^ cells were incubated with a high titer (MOI = 10) of influenza A/PR/8 (H1N1) virus such that almost all cells were infected and then the infection medium was removed. Unlike typical SCG experiments, however, viral titer was not measured by sacrificing independent wells at each sampling time to titrate their overlay. Instead, the liquid overlay from the same well was removed in its entirety and replaced with fresh, virus-free medium and the infection was allowed to continue. Thus, titrations of the collected overlay medium provided a measure of the amount of virus being produced by a single cell culture at the time of collection. Application of this sampling protocol to SCG experiments — which we refer to as a single-cycle, single-history yield experiment (SCSH) — mitigates complications tied to the accumulation of virus, and brings into focus the viral production of the cell culture as a series of snapshots over time, all sharing a common kinetic history.

**Figure 5:**
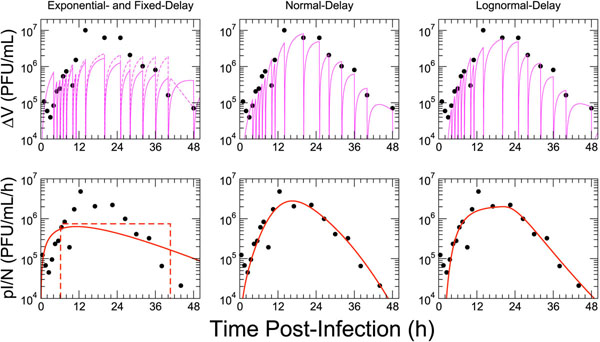
**Results of a single-cycle, single-history yield experiment**[9]**with model fits.Top row:** Experimentally-measured viral titers (points) are plotted along with model fits to the data (lines) for four different delay distributions. The left panel shows both the exponential-delay (solid) and fixed-delay (dashed) model fits. Discontinuities in the model dynamics result from removal of 99.9% of virus at each measurement time point, duplicating the experimental procedure. **Bottom row:** A transformation of the experimental data according to Equation (10) (points) shows the average viral production rate (i.e., the production rate weighted by the fraction of infectious cells) as a function of time; the model dynamics for same quantity, *pI/N*, are over-plotted (lines). The model dynamics plotted in the bottom row were generated using the extracted parameters from the fits performed to the raw data in the top row. While the exponential and fixed-delay models offer a poor fit to the data, both the normal and lognormal-delay models adequately describe the data.

Using the model in Equation (1), we performed fits to the SCSH data set [9] (Figure 5), allowing both the lengths of the latent and of the infectious periods to vary freely. To simulate the removal of the overlay medium at each sampling time, we reduced the virus concentration by a factor of 10^3^ (consideration of larger reductions showed a negligible influence on the fit). The first three data points were excluded from consideration while fitting since these points were likely due to the desorption of excess virus into the overlay [9], a process not accounted for in our model. The value of viral clearance was held fixed at 0.26 h^–1^, based on the results of an independent mock-infection experiment (Figure 7 of [9]). Four model types were considered: exponential-, fixed-, normal- and lognormal-delay. The best-fit virus curves are shown in Figure 5. The fitted parameter, SSR and AIC_c_ values for each distribution are reported in Table 3. As in SCG experiments, the normal-delay and lognormal-delay models provided adequate representations of the data, while the fixed-delay (DDE) and exponential-delay (ODE) models did not.

**Table 3:** Fits to the Gaush & Smith (1968) single-cycle, single-history experiment (Figure 5) [9]

Dist^a^		*τ_L_* (h)	*σ_L_*	*τ_I_* (h)	*σ_I_*	(h)	(h)	SSR	AIC_c_
exp	1.8 *×* 10^6^	10.5	—	10.5	—	7.3	7.3	3.59	-21.3
*δ*	7.5 *×* 10^5^	6.2	—	34.5	—	6.2	34.5	—^b^	—
*N*	5.4 *×* 10^6^	12.4	4.6 h	0.17	9.7 h	12.4	6.6	0.79	-41.3
	[1.4:8.7]^c^	[6.7:14.3]	[1.9:5.8]	[0.002:23]	[4.3:13.3]				
ln*N*	2.0 *×* 10^6^	8.5	0.49	18.2	0	8.5	18.2	0.93	-38.3
	[1.0:4.6]	[5.4:10.8]	[0.24:0.58]	[9.6:25.6]	[0:0.41]				

a — Delay model used: exponential (exp), fixed (*δ*), normal (*N*), and lognormal (ln*N*).

b — Using the fixed-delay model, virus titer is zero at all times prior to the appearance of infectious cells such that the SSR measured in log scale cannot be computed.

c — 95% confidence intervals are given in brackets for the normal and lognormal fits.

The unique attribute of the SCSH experiment is the view it provides of the viral *production* by the average infected cell over time. To investigate these kinetics, a transformation of the original data is required. Specifically, the experimental measure of accumulated virus ∆*V* over an interval ∆*t* can be converted to an average virus production rate for that time interval, i.e., the fraction of infectious cells multiplied by the (constant) viral production rate of a cell, *pI/N.* The theoretical relationship between these quantities is provided by the basic equation for viral dynamics, Equation (1d). If we assume that the number of infectious cells, *I,* remains constant over the interval between samplings of the overlay, this equation can be solved to obtain accumulated virus as a function of average production rate and viral clearance, *c*:(9)

where we have used the fact that the virus concentration at the beginning of the time interval is zero. Rearranging this expression, the average viral production over an interval ∆*t* centered at time *t* can then be written(10)

The resulting transformed data is shown in Figure 5. Viewed in this way, one can explicitly see the growth of the infectious cell population, including a steep rise from 4 h to 12 h as cells transition from latent to infectious, and a long decline between 24 h and 48 h as infectious cells cease viral production. The model dynamics for *pI/N,* generated using parameters from the fit to the raw data, agree well with the transformed data, validating the use of Equation (1d) and the assumptions made in the above transformation.

While the normal-delay and lognormal-delay models both lead to an adequate description of the experimental data, a consideration of the fitted parameter values for the median infectious cell lifespan, , reveals vastly different underlying dynamics. In the normal-delay case, the median infectious lifespan is predicted to be short  but the associated standard deviation is large. The long decay of infectious cells at late times is therefore explained by a broad distribution in the times spent by cells in the infectious state. Using the lognormal-delay model, however, the best fit is nearly that of a fixed-delay (*σ_I_* ≪ 1), with a long median infectious lifespan  Under this assumption, the decay of infectious cells is thus completely determined by the long tail in the distribution of *latently infected* cell lifespans. With only a single data set, it is not possible to discriminate between these two extreme cases: short infectious lifespans on average with a broad distribution, versus a long average infectious lifespan with a narrow distribution of transitions. The results of SCG experiments, where viral titer is observed to grow linearly over 10 to 20 h (Figure 3), suggest that the former is unlikely since infectious cell death would lead to a turnover in the viral titer curve. It would be useful to duplicate this unique experiment in parallel with a typical SCG experiment such that these biologically distinct possibilities can be distinguished.

### Effect of delay assumptions in fitting clinical data

**Figure 6:**
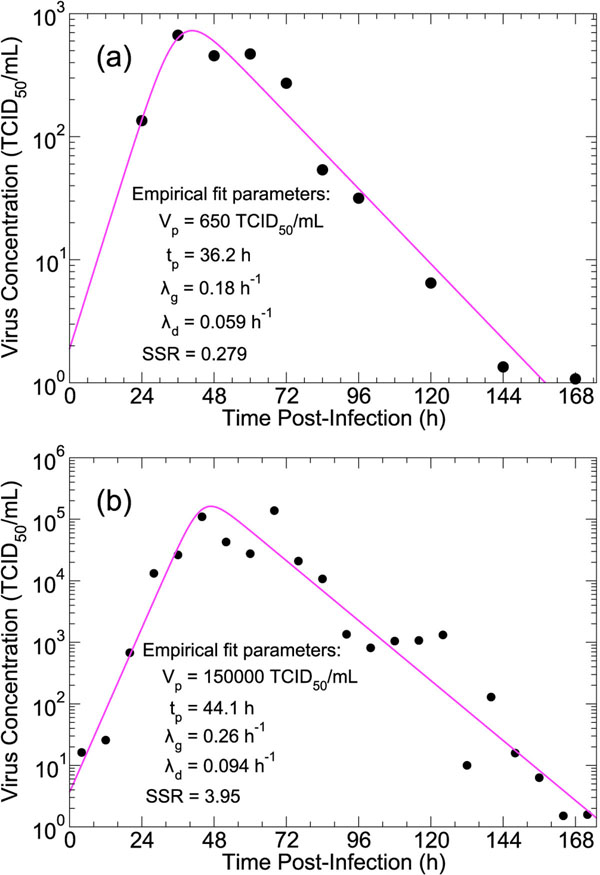
**In vivo patient data.** Viral titer over time for volunteer patients infected with (a) influenza A/Texas/36/91 (H1N1) [38], and (b) A/Bethesda/1/85 (H3N2) [39]. The solid curve is a fit of the empirical model in Equation (11) to each dataset. The fitted parameter values are given along with the SSR of the fit.

We now consider the effect that the choice of delay distribution has on estimated parameter values when fitting a model to viral titer data from human patients experimentally infected with influenza. This type of data — two examples are shown in Figure 6, for others see [34] — generally shows an exponential increase of virus followed by an exponential decrease after the peak, which occurs 1 to 2 d post-infection. An empirical function capturing these basic characteristics can be written as(11)

where *λ_g_* and *λ_d_* are the exponential growth and decay rates, respectively. *V_p_* is the peak value of the viral titer, and *t_p_* is the time of viral titer peak. The exact peak values can be determined by differentiating Equation (11). Fits of this function to the experimental data, along with the calculated SSR, are shown in Figure 6. The simple functional form of the empirical model in Equation (11) points to a fundamental problem in fitting a dynamical model to clinical viral data: only four independent parameters can be reliably extracted from such data. A closely related problem is whether or not the parameters of a given model can be uniquely identified from a particular experimental measurement, the question of parameter identifiability [35-37]. It has been shown, for example, that the ODE model in Equation (3) is not identifiable when considering viral titer data alone [37]. Sparse experimental data, where the viral titer curve is not well-sampled, can introduce additional complications for model fitting. For example, in the influenza A/Texas/36/91 (H1N1) viral titer data [38] presented in Figure 6a, there is only a single data point (at 24 h) from which the growth rate of viral titer, *λ_g_*, can be determined.

To mitigate some of these problems, we fitted the four delay models (exponential-, fixed-, normal- and lognormal-delay) to the clinical data sets in Figure 6 under a number of constraints. We fixed the values of *σ_L_* and *σ_I_* to those obtained for SCSH experiment (Table 3) to allow for comparison of model systems with an equal number of parameters. Then, to allow for unique solutions in the fitting algorithm, we reduced the parameter space by fixing the product of the viral production and infection rates, *pβ,* to the value of 1 h^–2^. This quantity is a measure of the infectivity of a virus-cell system and is related to the characteristic *infecting time* of the system,  which is time for a single infectious cell to cause the latent infection of one more in a completely susceptible cell population [33]. Under these constraints we were able to compare the effect of different delay assumptions on the fitted values of viral clearance, *c*, and production rate, *p* ; median latent and infectious cell lifespans, , and , and the basic reproductive number *R*_0_ (Table 4).

**Table 4:** Constrained model fits to the human influenza infection data in Figure 6.

A/Texas/36/91 (H1N1) [38]											
Dist^a^	*c* (h^–1^)		*p*^b,c^	*τ_L_* (h)	*σ_L_*	*τ_I_* (h)	*σ_I_*	(h)	(h)	*R*_0_	SSR^d^

exp	0.19	0.022	280	5.4	—	16.9	—	3.7	11.7	89	1.00
*δ*	0.059	2.85	62	10.2	—	23.0	—	10.2	23.0	390	1.00
*N*	0.059	0.013	100	1.8	(4.6 h)^c^	12.1	(9.7 h)	3.9	13.4	240	0.98
ln*N*	0.059	0.72	99	8.7	(0.49)	14.5	(0)	8.7	14.5	250	1.00

A/Bethesda/1/85 (H3N2) [39]											

Dist	*c* (h^–1^)	*V*_0_	*p*	*τ_L_* (h)	*σ_L_*	*τ_I_* (h)	*σ_I_*	(h)	(h)	*R*_0_	SSR

exp	0.11	7.0	1.3 *×* 10^5^	9.0	—	2.2	—	6.4	1.5	20	0.91
*δ*	0.10	28.4	7.7 *×* 10^3^	9.9	—	31.5	—	9.9	31.5	320	0.88
*N*	0.10	10.6	1.2 *×* 10^4^	9.6	(4.6 h)	19.9	(9.7 h)	9.7	20.1	200	0.90
ln*N*	0.10	11.8	8.0 *×* 10^3^	8.5	(0.49)	30.4	(0)	8.5	30.4	300	0.91

a — Delay model used: exponential (exp), fixed (*δ*), normal (*N*), and lognormal (ln*N*).

b — Units of *p* are [*V*]·h^–1^, where [*V*] = TCID_50_/mL, the units of viral titer.

c — The value of *pβ* was held fixed at 1 h^–2^ for all fits; values in parentheses were also eld fixed.

d — SSR′ is the ratio of the SSR to that of the empirical fit.

A comparison of the fitted parameter values shows a clear delineation between the results obtained under the assumption of exponentially-distributed delays and those of the other three models, which enforce longer delays for all cells. The median lifespan of a latently infected cell, , is shorter under the exponential assumption (3.7 h vs. an average of 7.6 h for the A/Texas/36/91 (H1N1) data set [38] and 6.4 h vs. an average of 9.4 h for A/Bethesda/1/85 (H3N2) [39]). The same is true for the median infectious lifetime, . Extracted values for the viral production rate are larger for the exponential-delay model, by a factor of ~ 2 in one data set and by an order of magnitude in the other. The estimated basic reproductive number is smaller for the exponential-delay model, by a factor of 2–10, than the other three delay types. The fitted value of the viral clearance, *c*, was independent of delay choice for one data set (and equal to the viral decay rate, *λ_d_*), but differed for the exponential-delay model in the other.

## Conclusions

Mathematical models of viral infections within a host or cell culture have helped shed light on several aspects of cell-virus interactions [6,40,41]. Most frequently, models have been used to extract values for the parameters controlling small-scale infection kinetics from experimental data [4,42,43]. This has allowed mathematical modeling to play an ever-growing role in virological and immunological studies, with an increasing number of publications in these fields incorporating some amount of modeling [44-46]. At present, however, modeling work often lags experimentation and primarily serves an explanatory role. It is desirable that models take a more predictive role in the future, where the simulation of viral infections may aid, for example, in the prediction of virulence for new strains or the kinetic mechanisms of untested antiviral therapies. Before models can take on a predictive role, however, the mathematical implementation of viral infection dynamics must be tested against a diverse set of experimental conditions to ensure that biological reality is faithfully represented.

Here, we have investigated the implementation of the progress of a cell through the states of infection: from latently infected to infectious to dead. We have focused on the characterization of two times: the time a cell is latently infected but not yet releasing virus, and the time a cell is infectious (releasing virus) before infection-induced death. We explored four different distributions for these state lifetimes: exponential, Dirac delta (fixed-delay), normal, and lognormal. The validity of each distribution was assessed by fitting the associated model to data from single-cycle growth (SCG) viral yield experiments. These experiments provide a unique view of the average dynamics of a single cell due to the synchronous infection of all cells. We have shown that ODE models which implement exponential delays and DDE models with fixed delays are unable to describe this experimental data, whereas normal-delay and lognormal-delay models both provide a good fit to the data. In addition to the classic SCG experiment, we have also considered delay dynamics for a “single-cycle, single-history” (SCSH) experiment [9], which reveals average cellular virus production as a function of time. We have shown that, like the SCG experiment, both normal- and lognormal-delay models provide an adequate description of the dynamics while exponential- and fixed-delay models do not. The origin of the inability of ODEs and DDEs to exhibit single-cycle dynamics can be seen quite clearly in the experimental data (Figures 3, 4, and 5): virus production begins only after a long delay following the infection of a cell, a feature which ODE models cannot replicate, and the transition of cells into and out of the infectious phase follows a smooth distribution which DDE models cannot reproduce.

The median values of the latent infection period, determined by fitting normal- and lognormal-delay models to the data from these SCG experiments, ranged from 8 h to 24 h, which is significantly longer than the 4 to 6 h values typically quoted in the literature (e.g., [2]). There are a number of possible reasons for this discrepancy. First, the latent infection period in the model includes both the typical eclipse period prior to virus production and any additional time required for viral release. Second, viral production in a culture is likely detectable much earlier. For example, under the assumption of normal delays the median length of the latent phase was found in the SCSH experiment to be  with *σ_L_* = 4.6 h. Thus, 6% of cells (~ 600*,* 000) had already begun releasing virus after only 6 h; using an alternative definition, the phase of latency could be declared over much earlier. Finally, the model describes a system where each cell releases no virus until the start of the infectious phase at which point virus is produced at a constant rate common to all cells. This is obviously a simplified version of reality. In fact, there is significant evidence from flow cytometry fluorescence experiments that different cells produce virus at different rates, perhaps over several orders of magnitude [47]; it is also likely that the rate of virus production varies over the course of its infectious lifespan.

The median infectious cell lifespan, determined in fits of the normal- and lognormal-delay models to the SCSH data, ranged from 6 to 18 h, but the application of these two distributions types implied disparate dynamical scenarios. Under the assumption of normal delays, the infectious lifespan was small but the distribution was broad. In contrast, the lognormal assumption predicted nearly a fixed infectious lifetime for all cells and the observed slow decline of infections cells was completely ascribed to a long tail in the transition from the latent to infectious phase. Biologically, the infectious cell lifespan is variously characterized in the literature, as is influenza-induced cell death in general. When cell death is due to apoptosis, for example in MDCK cell cultures [48], the median time of cell death ranges from 12 to 48 h after infection, depending on the influenza strain subtype. When virus-induced cell death is caused by necrosis, as in cultures of some lung and intestinal epithelial cells, this range increases significantly with cells living 2–3 d post-infection [20,49,50]. The death of cells during an infection in vivo is obviously much more difficult to measure. There is some evidence that influenza-induced cell death is caused by apoptosis [51-53] but details of the timing and strain dependence are unknown. Future elaboration of SCSH experiments, in concert with the type of analysis performed here, could aid in the quantitative characterization of virus-induced cell death.

Using the results from our analysis of in vitro experiments, we considered the effect of delay distribution choice when fitting viral titer data from in vivo infections (experiments performed on human volunteers). To allow for the identification of some kinetic parameters, we restricted the analysis by assuming a fixed value for the viral infectivity (defined as the product of the infection and production rates, *pβ*) and used the values for the parameters *σ_L_* and *σ_I_* determined when fitting the in vitro data. Under these assumptions, we found a clear difference between the extracted parameter values for models enforcing a delay in transitions (fixed-, normal- and lognormal-delay) and the ODE model with exponential transitions. Specifically the ODE predicted larger virus production rates, shorter latent and infectious phase lifespans, and a lower value of the basic reproductive number, *R*_0_ (Table 4). Although this result depends on rather arbitrary assumptions — infectivity may vary by orders of magnitude and there is little reason to assume that parameter values determined in vitro should be the same in vivo — it demonstrates that the choice of delay distribution has a significant effect on the conclusions drawn from model-fitting of in vivo data. This analysis also suggests that while a fixed-delay model cannot reproduce the continuous dynamics of cell transitions observed in single-cycle experiments, it may be a reasonable substitute for the more complicated normal and lognormal models in some contexts.

Our analysis of the in vivo experiments demonstrates the difficulty in extracting reliable information from viral titer data alone. Consideration of the SSR values for the model fits to in vivo data (Table 4) shows that each model adequately describes the data, despite the arbitrary constraints imposed. This highlights both the weakness of using viral titer data for model selection and the difficulty in uniquely identifying parameters from such data, given a particular model. Precise statements about the parameters controlling an in vivo infection can only be made by either imposing constraints to reduce the considered parameter space (as we have done here), or by obtaining complementary data (for example information about infectious cell population dynamics). Collection of infected cell data over the course of an influenza infection has only recently been considered in animal models (e.g., [54]) and such data will likely be unobtainable for human infections. Full characterization of models using in vitro experiments will therefore remain an important direction of future research. The introduction of infection models with an explicit immune response and the parallel measurements of immune system quantities (which are easier to obtain than infected cell populations) is a promising direction not only for a more complete understanding of the influenza infection but also the complete parametrization of these models [5,54,55] This will, of course, depend on the development of simple models for which the added complexity is warranted by the available data.

In the past ten years, tools for dynamical measurements of in vitro viral infections have improved quickly. The measurement of infection within individual cells by fluorescence microscopy has become routine [47]. The spatial spread of virus infections on cell culture can be viewed in real time, both at the level of cellular deformation and at the level of individual virus particles [56]. Individual virus particles have been tracked as they enter a cell, and repeated observations have allowed for a statistical characterization of the timing of events in the early stages of infection [16]. These detailed views of the influenza infection will be invaluable for the construction of the next generation of viral infection models. Models, in turn, will provide a cohesive picture of the overall infection process and, crucially, make connections between the known small-scale details of the virus-cell interaction and the infection at the level of the organism, where it is manifested as a disease.

## List of abbreviations

ODE: Ordinary differential equation; DDE: Delay differential equation; SSR: Sum of squared residuals; AIC: Akaike information criterion; AIC_c_: Akaike information criterion, corrected for small samples; SCG: Single-cycle growth; MOI: Multiplicity of infection; SCSH: Single-cycle, single-history growth; MDCK: Madin Darby canine kidney; VU: Virus units

## Competing interests

The authors declare that they have no competing interests.

## Authors' contributions

BH conceived the study, participated in the design of the study, carried out the modeling, performed the analysis and drafted the manuscript. CAAB conceived of the study, participated in its design and coordination, and helped to draft the manuscript. All authors read and approved the final manuscript.
